# Could sodium imbalances predispose to postoperative venous thromboembolism? An analysis of the NSQIP database

**DOI:** 10.1186/s12959-018-0165-5

**Published:** 2018-07-03

**Authors:** Sally Temraz, Hani Tamim, Aurelie Mailhac, Ali Taher

**Affiliations:** 1 Department of Internal Medicine, American University of Beirut Medical Center, Riad El Solh 110 72020, Beirut, Lebanon; 2 Clinical Research Institute, American University of Beirut Medical Center, Beirut, Lebanon

**Keywords:** Hypernatremia, Hyponatremia, Mortality, Morbidity, Surgery, Venous thromboembolism

## Abstract

**Background:**

Hyponatremia is common among patients with pulmonary embolism, while hypernatremia increases the risk of venous thromboembolism (VTE). Our objective was to evaluate the association between sodium imbalances and the incidence of VTE and other selected perioperative outcomes.

**Methods:**

We conducted a retrospective cohort study using the American College of Surgeons National Surgical Quality Improvement Program (ACS NSQIP) and identified 1,108,704 patients undergoing major surgery from 2008 to 2012. We evaluated 30-day perioperative outcomes, including mortality and cardiac, respiratory, neurological, urinary, wound, and VTE outcomes. Multivariate logistic regressions were used to estimate the odds of 30-day perioperative outcomes.

**Results:**

Compared with the normal sodium group, in which VTE occurred in 1.0% of patients, 1.8% of patients in the hyponatremia group (unadjusted odds ratio (OR) 1.84) and 2.4% of patients in the hypernatremia group (unadjusted OR 2.49) experienced VTE. Crude mortality was 1.3% in the normal sodium group, 4.9% in the hyponatremia group (unadjusted OR 3.93) and 8.4% in the hypernatremia group (unadjusted OR 7.01). Crude composite morbidity was 7.1% for the normal sodium group, 16.7% for the hyponatremia group (unadjusted OR 2.63) and 20.6% for the hypernatremia group (unadjusted OR 3.43). After adjusting for potential confounders, hyponatremia and hypernatremia remained significantly and independently associated with an increased risk of VTE (adjusted OR 1.43 and 1.56, respectively), mortality (adjusted OR 1.39 and 1.39, respectively) and composite morbidity (adjusted OR 2.15 and 3.34, respectively).

**Conclusions:**

Pre-operative hyponatremia and hypernatremia are potential prognostic markers for perioperative 30-day morbidity, mortality and VTE.

## Background

Hyponatremia is the most common electrolyte imbalance in hospitalized patients, afflicting at least 30% of patients in medical, surgical and psychiatric wards [1, 2]. Hyponatremia is a complicated condition that has been associated with all-cause mortality [3]; length of inpatient stay [4]; gait and attention impairments [5]; bone fractures [6, 7]; and perioperative complications, including 30-day morbidity and mortality [8, 9]. Most studies that have evaluated the outcomes associated with hyponatremia restricted their analyses to hospitalized patients with pre-existing medical conditions, such as congestive heart failure, chronic kidney disease and liver cirrhosis. Although hypernatremia is also associated with increased mortality, most studies have focused on hyponatremia [4]. Hyponatremia is common in patients presenting with acute pulmonary embolism [10, 11], while hypernatremia is associated with increased risk of venous thromboembolism (VTE) [12]. To date, no study has evaluated the association between hyponatremia and the occurrence of thromboembolic events.

VTE includes both deep vein thrombosis and pulmonary embolism and is a major cause of morbidity and mortality worldwide. Approximately 1 in 1000 adults is affected by VTE annually, with the incidence increasing with age [13]. VTE ranks third among cardiovascular diseases, preceded only by coronary artery disease and cerebrovascular disease [14]. The incidence of VTE is increased 100-fold higher in hospitalized patients than in the general population [15], and VTE is detected in approximately 80% of surgical and medical patients who are not placed on thromboprophylaxis [16].

Our objective was to perform a population-based retrospective cohort study of patients in the American College of Surgeons National Surgical Quality Improvement Program (ACS NSQIP) database to evaluate the association between sodium imbalances and the incidence of VTE and other selected perioperative outcomes.

## Methods

### Study design and data collection

The ACS NSQIP is a nationally validated, outcome-based, risk-adjusted program developed to improve the quality of surgical care for adults in the United States. The program prospectively collects > 150 variables, including pre- and intraoperative variables and 30-day postoperative mortality and morbidity outcomes for patients undergoing major outpatient or inpatient surgical procedures in more than 200 participating non-Veterans Affairs administration hospitals [17, 18]. At each hospital site, a trained and certified Surgical Clinical Reviewer (SCR) captures these data using a variety of methods including medical chart abstraction and International Classification of Diseases (ICD) coding. The accuracy of the outcomes of NSQIP dataset are ensured by a host of different training mechanisms for the SCRs and by an Inter-Rater Reliability (IRR) Audit of selected participating sites. Based on the ACS NSQIP participant use files from 2008 to 2012, we identified 1,957,023 adult patients (aged ≥18 years) who underwent major surgery (defined by Current Procedural Terminology codes), No patient was included in the database twice, and only the index case was used for patients who had undergone more than one procedure. We excluded patients who were pregnant and who did not have their sodium levels recorded in their files. Also, due to the high percentages of missing values in a set of confounders, patients with missing values on this set of variables were excluded. A total of 1,108,704 patients were included in the analyses. In accordance with the American University of Beirut’s guidelines, which follow the US Code of Federal Regulations for the Protection of Human Subjects, institutional review board approval was not needed or sought for our analysis because the data were collected as part of a quality assurance activity.

### Procedures

Our exposure of interest was the most recent serum sodium measurement within 90 days of surgery. Patients were assigned to one of three groups based on their pre-operative sodium levels. Hyponatremia was defined as sodium < 135 mEq/L, normal sodium levels were between 135 and 145 mEq/L, and hypernatremia was defined as > 145 mEq/L. The primary postoperative outcomes were incidences of VTE (defined as the presence of DVT and/or PE during the postoperative period) and 30-day mortality. DVT is currently defined within ACS NSQIP as the “identification of a new blood clot or thrombus within the venous system”, which may be coupled with inflammation within 30 days of the operation. This diagnosis is confirmed by a duplex (ultrasound), venogram or CT scan. The patient must be treated with anticoagulation therapy or placement of a vena cava filter or clipping of the vena cava [19]. PE is defined as “lodging of a blood clot in a pulmonary artery with subsequent obstruction of blood supply to the lung parenchyma”. PE is documented if the patient has a V-Q scan interpreted as high probability of PE or a positive CT spiral exam, pulmonary arteriogram or CT angiogram [19]. 30-day mortality is defined as any cause of death (intraoperative or postoperative) occurring within 30 days of surgery. Secondary outcomes included composite comorbidities (wound, cardiac, respiratory, urinary, central nervous system (CNS), sepsis, bleeding, return to operation room and hospital readmission.

### Statistical analysis

Statistical analysis was performed using SAS (SAS version 9.1; SAS, Inc., Cary, NC). *P*-values were 2-sided, and significance was set at 0.05. Baseline demographics and pre-operative and perioperative variables were described for all three categories of blood sodium. Differences were analyzed across categories using the chi-square test for categorical variables and ANOVA for continuous variables. Separate simple and multivariate logistic regression models were used to evaluate the association between blood sodium levels and each outcome. The reference category for the main exposure was set as the normal blood sodium levels. Adjusted odds ratios (ORs) were estimated by including clinically relevant potential confounders of individual outcomes in the models, as outlined in Appendix. Stepwise regression was performed at an entry level of 0.15 and a stay level of 0.25. To assess effect modification between sodium and VTE, adjusted ORs were estimated by stratifying for age, sex, surgical specialty, steroid use for chronic conditions, body mass index (BMI), presence of active cancer and chemotherapy.

## Results

For the 1,108,704 patients in this study, the mean age was 57.70 years (SD = 16.69), and 56.2% of patients were female. Sodium levels were normal in 1,010,167 patients, 87,476 had hyponatremia, and 11,061 had hypernatremia. Table 1 presents demographics and baseline patient characteristics for each of the sodium groups. Compared with patients with normal sodium levels, patients with hyponatremia and hypernatremia were more likely to be older, inpatients, in a high American Society of Anesthesiologists (ASA) class, exposed to prolonged anesthesia, emergency cases, and under a “do not resuscitate” status. Additionally, these patients were more likely to have partially or totally dependent functional status, have lost more than 10% of their body weight in the prior 6 months, have abnormal pre-operative laboratory studies, and receive perioperative transfusions. Patients with hyponatremia and hypernatremia also had a higher prevalence of dyspnea, diabetes, systemic sepsis, and cardiovascular, respiratory, hepatobiliary, renal, neurological, and hematological-oncological disorders; chronic steroid use; operations within the past month; and infected surgical wounds (Table 1). Patients with normal sodium levels were more likely to be younger than 50 years of age, undergo gynecological, orthopedic, urological and plastic surgeries, have an independent functional status and have a BMI > 35. Patients with hyponatremia were more likely to be male; have undergone thoracic, vascular and cardiac surgeries; have smoked within 1 year; drink more than two drinks per day; and have a BMI < 35. Conversely, patients with hypernatremia were more likely to be non-white, have undergone general and otolaryngology surgeries, have undergone general anesthesia and have been in a coma for more than 24 h (Table 1).

**Table 1:** Baseline patient characteristics across three categories of blood sodium levels

	Sodium (mEq/L)			
	< 135	135–145	> 145	*p*-value
	(*n* = 87,476)	(*n* = 1,010,167)	(*n* = 11,061)	
Age	61.82 ± 16.52	57.29 ± 16.65	63.38 ± 15.89	< 0.0001
< 50	19,723 (22.6)	316,094 (31.3)	2015 (18.2)	< 0.0001
50–64	26,535 (30.3)	330,181 (32.7)	3500 (31.6)	
65–79	27,788 (31.8)	271,429 (26.9)	3653 (33.0)	
≥ 80	13,430 (15.4)	92,463 (9.2)	1893 (17.1)	
Sex, female	44,389 (50.9)	570,528 (56.6)	6142 (55.6)	< 0.0001
Race, White	66,224 (84.8)	759,216 (84.6)	7777 (78.0)	< 0.0001
Surgery type				
General surgery	54,423 (62.2)	656,018 (64.9)	7529 (68.1)	< 0.0001
Gynecology	1503 (1.7)	35,143 (3.5)	242 (2.2)	
Neurosurgery	2234 (2.6)	26,292 (2.6)	262 (2.4)	
Orthopedics	6831 (7.8)	87,304 (8.6)	782 (7.1)	
Otolaryngology (ENT)	922 (1.1)	15,953 (1.6)	206 (1.9)	
Plastics	615 (0.7)	12,522 (1.2)	150 (1.4)	
Thoracic	1289 (1.5)	10,221 (1.0)	128 (1.2)	
Urology	2400 (2.7)	39,573 (3.9)	361 (3.3)	
Vascular	15,853 (18.1)	116,611 (11.5)	1317 (11.9)	
Cardiac surgery	1404 (1.6)	10,524 (1.0)	83 (0.8)	
Principal anesthesia technique, general	80,842 (92.4)	929,863 (92.1)	10,238 (92.6)	< 0.0001
ASA classification				
I-II	25,653 (29.4)	512,018 (50.8)	3642 (33.0)	< 0.0001
III	45,074 (51.7)	422,264 (41.9)	4666 (42.3)	
IV-V	16,531 (18.9)	73,419 (7.3)	2721 (24.7)	
Wound classification, dirty/infected	17,210 (19.7)	59,804 (5.9)	1163 (10.5)	< 0.0001
Inpatient status	74,600 (85.3)	704,245 (69.7)	8451 (76.4)	< 0.0001
Emergency case	25,053 (28.6)	123,927 (12.3)	2396 (21.7)	< 0.0001
Transfusion in 72 h before surgery	1992 (2.3)	8644 (0.9)	643 (5.8)	< 0.0001
Do not resuscitate (DNR) status	1601 (1.8)	6425 (0.6)	306 (2.8)	< 0.0001
Functional health status before surgery				
Independent	73,530 (84.2)	952,051 (94.4)	8620 (78.1)	< 0.0001
Partially dependent	10,052 (11.5)	42,984 (4.3)	978 (8.9)	
Totally dependent	3708 (4.3)	14,080 (1.4)	1439 (13.0)	
Dyspnea	13,284 (15.2)	106,592 (10.6)	1755 (15.9)	< 0.0001
CHF 30 days before surgery	2530 (2.9)	9234 (0.9)	443 (4.0)	< 0.0001
History of angina 1 month before surgery	1579 (1.8)	10,498 (1.0)	163 (1.5)	< 0.0001
History of MI 6 months before surgery	1650 (1.9)	7518 (0.7)	267 (2.4)	< 0.0001
Previous PCI	7353 (8.4)	61,587 (6.1)	881 (8.0)	< 0.0001
Previous cardiac surgery	8133 (9.3)	59,855 (5.9)	948 (8.6)	< 0.0001
Hypertension requiring medication	55,678 (63.7)	510,100 (50.5)	6682 (60.4)	< 0.0001
History of revascularization/amputation for peripheral vascular disease	8298 (9.5)	40,414 (4.0)	580 (5.2)	< 0.0001
Smoking within 1 year	22,555 (25.8)	197,346 (19.5)	2045 (18.5)	< 0.0001
Current pneumonia	1537 (1.8)	4815 (0.5)	514 (4.7)	< 0.0001
History of severe COPD	8372 (9.6)	52,570 (5.2)	967 (8.7)	< 0.0001
Ventilator dependent	1926 (2.2)	7756 (0.8)	1193 (10.8)	< 0.0001
Ascites	2427 (2.8)	6325 (0.6)	286 (2.6)	< 0.0001
Esophageal varices	302 (0.4)	1017 (0.1)	27 (0.2)	< 0.0001
Acute renal failure	1800 (2.1)	5066 (0.5)	336 (3.0)	< 0.0001
Currently on dialysis	4758 (5.4)	18,196 (1.8)	353 (3.2)	< 0.0001
Impaired sensorium	2018 (2.3)	6524 (0.7)	660 (6.0)	< 0.0001
Coma > 24 h	136 (0.2)	596 (0.1)	99 (0.9)	< 0.0001
History of transient ischemic attacks (TIA)	3662 (4.2)	31,358 (3.1)	421 (3.8)	< 0.0001
CVA/stroke with neurological deficit	3432 (3.9)	24,497 (2.4)	630 (5.7)	< 0.0001
CVA/stroke with no neurological deficit	2941 (3.4)	21,936 (2.2)	345 (3.1)	< 0.0001
Tumor involving CNS	498 (0.6)	3703 (0.4)	62 (0.6)	< 0.0001
Bleeding disorders	9989 (11.4)	57,672 (5.7)	1195 (10.8)	< 0.0001
> 10% loss body weight in previous 6 months	4158 (4.8)	19,804 (2.0)	376 (3.4)	< 0.0001
Disseminated cancer	3694 (4.2)	21,246 (2.1)	329 (3.0)	< 0.0001
Chemotherapy ≤30 days pre-operative	2051 (2.3)	14,897 (1.5)	239 (2.2)	< 0.0001
Radiotherapy in last 90 days	1011 (1.2)	7705 (0.8)	100 (0.9)	< 0.0001
BMI	28.40 ± 8.15	30.31 ± 8.42	29.57 ± 8.30	< 0.0001
< 18.5	4085 (4.9)	20,342 (2.1)	343 (3.2)	< 0.0001
18.5–24.9	27,699 (33.1)	249,205 (25.3)	2895 (27.3)	
25.0–29.9	24,643 (29.4)	300,443 (30.5)	3209 (30.3)	
30.0–34.9	13,555 (16.2)	192,841 (19.6)	2068 (19.5)	
≥ 35.0	13,716 (16.4)	221,151 (22.5)	2083 (19.7)	
Diabetes mellitus with oral agents or insulin	23,275 (26.6)	167,543 (16.6)	2174 (19.7)	< 0.0001
Alcohol > 2 drinks/day 2 wks before admission	4925 (5.6)	25,735 (2.6)	323 (2.9)	< 0.0001
Open wound/wound infection	11,046 (12.6)	43,031 (4.3)	1120 (10.1)	< 0.0001
Steroid use for chronic condition	4717 (5.4)	32,860 (3.3)	591 (5.3)	< 0.0001
Systemic sepsis	21,684 (25.0)	72,666 (7.3)	2411 (21.9)	< 0.0001
Prior operation within 30 days	6239 (7.1)	25,921 (2.6)	960 (8.7)	< 0.0001

Thromboembolism occurred in 1.0% of patients in the normal sodium group compared with 1.8% in the hyponatremia group (unadjusted OR 1.89, 95% CI 1.79–2.00) and 2.5% in the hypernatremia group (unadjusted OR 2.64, 95% CI 2.34–2.98) (Table 2). Crude mortality was 1.4% for the normal sodium group compared with 5.1% for the hyponatremia group (unadjusted OR 3.85, 95% CI 3.72–3.98) and 9.2% for the hypernatremia group (unadjusted OR 7.20, 95% CI 6.73–7.69) (Table 2). Crude composite morbidity was 7.3% for the normal sodium group compared with 16.9% for the hyponatremia group (unadjusted OR 2.59, 95% CI 2.54–2.64) and 22.1% for the hypernatremia group (unadjusted OR 3.61, 95% CI 3.45–3.78) (Table 2). Bleeding occurred in 4.3% of patients with normal sodium levels compared with 9.3% in those with hyponatremia (unadjusted OR 2.30, 95% CI 2.24–2.36) and 9.4% in those with hypernatremia (unadjusted OR 2.32, 95% CI 2.18–2.48). Of patients with normal sodium levels, 4.5% had subsequent surgery, and 5.9% were readmitted; by comparison, among patients with hyponatremia, these values were 9.7% (unadjusted OR 2.30, 95% CI 2.25–2.36) and 9.0% (unadjusted OR 1.59, 95% CI 1.52–1.67), respectively; and they were 9.1% (unadjusted OR 2.14, 95% CI 2.00–2.28) and 6.7% (unadjusted OR 1.15, 95% CI 1.00–1.33), respectively, for patients with hypernatremia (Table 2).

**Table 2:** Unadjusted analyses for associations between blood sodium levels and outcomes

				Unadjusted ORs		
	Sodium (mEq/L)			Sodium (mEq/L)		
	< 135	135–145	> 145	< 135	135–145	> 145
	(*n* = 87,476)	(*n* = 1,010,167)	(*n* = 11,061)	(*n* = 87,476)	(*n* = 1,010,167)	(*n* = 11,061)
Thromboembolism	1577 (1.8)	9705 (1.0)	276 (2.5)	1.89 (1.79–2.00)	Reference	2.64 (2.34–2.98)
Mortality	4479 (5.1)	13,972 (1.4)	1014 (9.2)	3.85 (3.72–3.98)	Reference	7.20 (6.73–7.69)
Composite morbidity^a^	14,795 (16.9)	73,563 (7.3)	2442 (22.1)	2.59 (2.54–2.64)	Reference	3.61 (3.45–3.78)
Wound	3908 (4.5)	23,316 (2.3)	439 (4.0)	1.98 (1.91–2.05)	Reference	1.75 (1.59–1.93)
Cardiac	1694 (1.9)	7472 (0.7)	263 (2.4)	2.65 (2.51–2.80)	Reference	3.27 (2.89–3.70)
Respiratory	7772 (8.9)	31,741 (3.1)	1639 (14.8)	3.01 (2.93–3.08)	Reference	5.36 (5.08–5.66)
Urinary	1724 (2.0)	7627 (0.8)	378 (3.4)	2.64 (2.51–2.79)	Reference	4.65 (4.19–5.17)
CNS	655 (0.8)	3938 (0.4)	135 (1.2)	1.93 (1.77–2.10)	Reference	3.16 (2.66–3.75)
Sepsis	5618 (6.4)	25,568 (2.5)	870 (7.9)	2.64 (2.57–2.72)	Reference	3.29 (3.06–3.53)
Bleeding	8128 (9.3)	43,090 (4.3)	1036 (9.4)	2.30 (2.24–2.36)	Reference	2.32 (2.18–2.48)
Return to operation room	8490 (9.7)	45,091 (4.5)	1004 (9.1)	2.30 (2.25–2.36)	Reference	2.14 (2.00–2.28)
Readmission (related)^b^	2062 (9.0)	16,099 (5.9)	203 (6.7)	1.59 (1.52–1.67)	Reference	1.15 (1.00–1.33)

^a^Composite morbidity considered positive if any of the following are present: wound, cardiac, respiratory, urinary, CNS injury, sepsis or thromboembolism

^b^Sample size: 300,815

After adjusting for potential confounders, hyponatremia was significantly and independently associated with an increased risk of thromboembolism (adjusted OR 1.43, 95% CI 1.36–1.52), mortality (adjusted OR 1.39, 95% CI 1.34–1.45), composite morbidity (adjusted OR 2.15, 95% CI 2.11–2.19), major bleeding (adjusted OR 1.96, 95% CI 1.91–2.01), return to operation room (adjusted OR 1.46, 95% CI 1.42–1.50) and readmission (adjusted OR 1.21, 95% CI 1.15–1.27) (Table 3). Hypernatremia was also significantly and independently associated with an increased risk of thromboembolism (adjusted OR 1.57, 95% CI 1.38–1.78), mortality (adjusted OR 1.39, 95% CI 1.27–1.52), composite morbidity (adjusted OR 3.33, 95% CI 3.18–3.49), major bleeding (adjusted OR 2.0, 95% CI 1.87–2.13), and return to operation room (adjusted OR 0.92, 95% CI 0.80–1.06) (Table 3).

**Table 3:** Adjusted analyses for associations between blood sodium levels and outcomes

	Sodium (mEq/L)		
	< 135	135–145	> 145
	(*n* = 87,476)	(*n* = 1,010,167)	(*n* = 11,061)
Thromboembolism	1.43 (1.36–1.52)	Reference	1.57 (1.38–1.78)
Mortality	1.39 (1.34–1.45)	Reference	1.39 (1.27–1.51)
Composite morbidity^a^	2.15 (2.11–2.19)	Reference	3.33 (3.18–3.49)
Wound	1.27 (1.22–1.32)	Reference	1.25 (1.13–1.38)
Cardiac	1.24 (1.17–1.31)	Reference	1.38 (1.21–1.57)
Respiratory	1.77 (1.72–1.82)	Reference	1.93 (1.80–2.07)
Urinary	2.28 (2.16–2.40)	Reference	3.96 (3.56–4.40)
CNS	1.45 (1.33–1.57)	Reference	2.25 (1.89–2.68)
Sepsis	1.72 (1.67–1.78)	Reference	2.11 (1.96–2.28)
Bleeding	1.96 (1.91–2.01)	Reference	2.00 (1.87–2.13)
Return to operation room	1.46 (1.42–1.50)	Reference	1.39 (1.30–1.49)
Readmission (related)^b^	1.21 (1.15–1.27)	Reference	0.92 (0.80–1.06)

^a^Composite morbidity considered positive if any of the following are present: wound, cardiac, respiratory, urinary, CNS injury, sepsis or thromboembolism

^b^Sample size: 300,815

The effect of hyponatremia on thromboembolic outcome was evident across all age groups, both sexes, non-orthopedic patients, patients with or without steroid treatment, patients with a BMI > 18.5, patients with or without cancer and patients with or without chemotherapy (Table 4). The effect of hypernatremia on thromboembolic outcome was evident across all age groups, both sexes, non-orthopedic patients, patients not on steroids, patients with a BMI > 18.5, patients with or without cancer and patients not receiving chemotherapy (Table 4).

**Table 4:** Stratified analyses for associations between blood sodium levels and VTE outcome

	Sodium (mEq/L)		
	< 135	135–145	> 145
	(*n* = 87,476)	(*n* = 1,010,167)	(*n* = 11,061)
Thromboembolism	1.43 (1.36–1.52)	Reference	1.57 (1.38–1.78)
Age			
< 50 (*n* = 337,832)	1.68 (1.45–1.94)	Reference	1.65 (1.14–2.38)
50–64 (*n* = 360,216)	1.65 (1.49–1.83)	Reference	1.42 (1.11–1.82)
65–79 (*n* = 302,870)	1.28 (1.17–1.41)	Reference	1.67 (1.37–2.03)
≥ 80 (*n* = 107,786)	1.17 (1.02–1.33)	Reference	1.40 (1.06–1.83)
Sex			
Male (*n* = 484,893)	1.37 (1.27–1.48)	Reference	1.54 (1.29–1.84)
Female (*n* = 623,811)	1.48 (1.36–1.60)	Reference	1.57 (1.31–1.88)
Surgical specialty			
Non-orthopedic (*n* = 1,013,787)	1.47 (1.39–1.56)	Reference	1.62 (1.43–1.85)
Orthopedic (*n* = 94,917)	1.10 (0.89–1.37)	Reference	0.71 (0.34–1.51)
Steroid use for chronic condition			
No (*n* = 1,070,536)	1.45 (1.37–1.54)	Reference	1.66 (1.46–1.89)
Yes (*n* = 38,168)	1.26 (1.06–1.51)	Reference	0.86 (0.53–1.39)
BMI			
< 18.5 (*n* = 24,770)	1.28 (1.00–1.63)	Reference	1.68 (0.92–3.07)
18.5–24.9 (*n* = 279,799)	1.51 (1.36–1.66)	Reference	1.52 (1.19–1.95)
25.0–29.9 (*n* = 328,295)	1.44 (1.30–1.60)	Reference	1.50 (1.17–1.93)
30.0–34.9 (*n* = 238,890)	1.30 (1.15–1.48)	Reference	1.45 (1.12–1.89)
≥ 35.0 (*n* = 236,950)	1.52 (1.32–1.74)	Reference	1.82 (1.39–2.38)
Presence of active cancer^a^			
No (*n* = 1,068,670)	1.42 (1.34–1.51)	Reference	1.53 (1.34–1.74)
Yes (*n* = 40,034)	1.25 (1.04–1.49)	Reference	1.55 (1.29–1.85)
Chemotherapy for malignancy ≤30 days pre-surgery			
No (*n* = 1,091,517)	1.42 (1.34–1.51)	Reference	1.57 (1.38–1.78)
Yes (*n* = 17,187)	1.67 (1.29–2.16)	Reference	1.57 (0.79–3.13)

^a^Considered positive if any of the following are present: disseminated cancer, tumor involving CNS or chemotherapy for malignancy ≤30 days pre-surgery

The median time from blood draw to surgery was 4 days. Because sodium levels vary over time, we performed a restricted sensitivity analysis for patients in whom blood was drawn within 1 week before surgery. Hyponatremia remained significantly and independently associated with an increased risk of thromboembolism, mortality, composite morbidity, major bleeding, return to operation room and readmission (Table 5). Hypernatremia also remained significantly and independently associated with an increased risk of thromboembolism, mortality, composite morbidity, major bleeding and return to operation room.

**Table 5:** Sensitivity analysis of outcomes for Na levels taken ≤7 days prior to surgery

	Sodium (mEq/L)		
	< 135	135–145	> 145
	(*n* = 71,783)	(*n* = 646,416)	(*n* = 7795)
Thromboembolism	1.40 (1.32–1.49)	Reference	1.66 (1.46–1.89)
Mortality	1.36 (1.30–1.41)	Reference	1.40 (1.28–1.53)
Composite morbidity^a^	2.04 (2.00–2.08)	Reference	3.86 (3.67–4.06)
Wound	1.27 (1.22–1.32)	Reference	1.28 (1.15–1.43)
Cardiac	1.24 (1.17–1.31)	Reference	1.42 (1.24–1.62)
Respiratory	1.67 (1.62–1.72)	Reference	1.96 (1.82–2.11)
Urinary	2.07 (1.96–2.19)	Reference	4.22 (3.78–4.70)
CNS	1.36 (1.24–1.49)	Reference	2.34 (1.95–2.81)
Sepsis	1.65 (1.59–1.70)	Reference	2.20 (2.04–2.38)
Bleeding	1.81 (1.76–1.86)	Reference	2.10 (1.96–2.26)
Return to operation room	1.43 (1.39–1.47)	Reference	1.47 (1.37–1.58)
Readmission (related)^b^	1.19 (1.12–1.25)	Reference	0.87 (0.73–1.03)

^a^Composite morbidity considered positive if any of the following are present: wound, cardiac, respiratory, urinary, CNS injury, sepsis or thromboembolism

^b^Sample size: 189,828

## Discussion

In this study, we assessed pre-operative hypo- and hypernatremia in patients across all surgical specialties by analyzing data from the ACS NSQIP database. Hyponatremia and hypernatremia were both significantly and independently associated with postoperative thromboembolism, mortality, morbidity, major bleeding and return to operation room. Only hyponatremia was associated with hospital readmission.

The reported incidence of hyponatremia upon hospital admission ranges from 5 to 30% depending on the study population and timing of serum sodium measurements [20–23]. We report a 7.89% rate of pre-operative hyponatremia among surgical patients. Hyponatremia has been associated with increased mortality in patients with pre-existing acute kidney injury [24], chronic kidney disease [25], heart failure [26–29], COPD [30], hip fractures [31], and intracerebral hemorrhage [32]; in patients undergoing cardiac transplantation [33]; and in unselected inpatients with hyponatremia [9, 34, 35]. The reported mortality rate ranges from 5.2 to 22% [8, 21, 22, 34, 36]. Our study had a 5.12% mortality rate among hyponatremic patients, similar to that reported by Leung et al. (5.2%), Waikar et al. (5.4%) and Zilberger et al. (5.9%). However, our mortality rate was lower than that reported by Holland-Bill et al. (8.1%) and much lower than that reported by Sturdik et al. (22%). The higher mortality rates reported by Holland-Bill et al. and Sturdik et al. may be related to their study populations, which included patients who were admitted to the internal medicine department. In contrast, our study population and that of Leung et al. involved surgical patients, and studies by Zilberberg et al. and Waiker et al. included a more general patient population. Increased morbidity [8, 28, 30] and 30-day hospital readmissions [26, 37] have also been reported in patients with hyponatremia. Hyponatremia is a common finding among patients with pulmonary embolism, occurring at rates ranging from 21 to 26% [10, 11]. In their meta-analysis, Zhou XY et al. reported that in-hospital mortality was 12.9% in hyponatremic patients with pulmonary embolism and 2.3% in normonatremic patients. The mean 30-day mortality was 15.9% in the hyponatremia group and was 7.4% in the normonatremia group [38]. However, no study has evaluated the direct association between hyponatremia and the development of pulmonary embolism or deep vein thrombosis.

Similar to hyponatremia, hypernatremia was associated with increased mortality in patients undergoing percutaneous endoscopic gastrostomy [39], cardiothoracic surgery [40], or cardiac transplantation; in hip fracture patients [31]; in patients with chronic kidney disease [25]; in critically ill patients [41–43]; and in patients with traumatic brain injuries [33, 44]. The reported mortality rate varies greatly, ranging from as low as 5.2% to as high as 82% [45–54]. This discrepancy is mainly due to data from elderly patients [46, 47, 49, 50] and on which cutoff value is used to define hypernatremia [51–54]. However, using a study population similar to ours, Leung et al. reported hypernatremia in 2.2% of surgical patients compared with 1% in our population and a mortality rate of 5.2%, which is close to our 9.16% mortality rate [12]. The authors similarly reported that hypernatremia predicted the occurrence of perioperative major coronary events, pneumonia, and VTE [12]. Our analysis was extended to evaluate the association of both hyponatremia and hypernatremia with the occurrence of thromboembolic events.

Postoperative thromboembolism within a time frame of 30 days occurred in 2.49% of patients with hypernatremia and 1.8% of patients with hyponatremia. Leung et al. reported thromboembolism among 1.8% of hypernatremic patients [12]. We found that the effect of hyponatremia on thromboembolic outcome was evident across all age groups, both sexes, patients with or without steroid treatment, patients with a BMI > 18.5, patients with or without cancer and patients with or without chemotherapy. However, hyponatremia was not associated with thromboembolic events in orthopedic patients. The effect of hypernatremia on thromboembolic outcome was also evident across all age groups, both sexes, patients with a BMI > 18.5, and patients with or without cancer. However, hypernatremic patients undergoing orthopedic surgery and patients on chemotherapy and steroids did not develop thromboembolisms. VTE is a serious complication following major orthopedic surgery, and all patients undergoing orthopedic surgery receive thromboprophylaxis with a pharmacological agent or Intermittent Pneumatic Compression Device (IPCD) for a minimum of 10 to 14 days, sometimes extending prophylaxis up to 35 days [55]. This prophylaxis could explain why hyponatremic and hypernatremic patients undergoing orthopedic surgery were not at risk of developing VTE. Systemic glucocorticoid use increases the risk of VTE among present, new, continuing and recent users but not among former users [56]. Corticosteroid therapy is associated with a nearly 5-fold increase in the risk of VTE [57]. Surgical patients with prolonged pre-operative glucocorticoid intake are at a higher risk of developing postoperative VTE and secondary outcomes, including all-cause mortality, urinary tract complications sepsis, wound occurrences, cardiac and respiratory adverse events [58]. This observation suggests that hyponatremic and hypernatremic patients on steroids are at risk for VTE. However, hypernatremic patients on steroids did not show an increased risk of VTE in our study. Hypernatremia is sometimes encountered in patients with hypertension secondary to aldosteronism [59]. Medical treatment involves the use spironolactone (aldosterone antagonist or Amiloride) in addition to angiotensin-converting enzyme inhibitors for better control of blood pressure [60]. Chae et al. recently reported that after controlling for factors related to VTE, the use of renin-angiotensin inhibitors was still associated with a significantly lower risk of developing VTE [61], which may explain why hypernatremic patients in our study receiving steroids were protected against VTE.

Chemotherapy use in cancer patients increases the risk of VTE 6.5-fold compared with non-cancer patients [62]. Salahudeen et al. recently reported that 90% of hypernatremia cases among cancer patients are hospital acquired and largely involve leukemia and stem cell transplant patients. The authors also found that, compared with patients with normo- or hyponatremia, patients with hypernatremia were extremely sick and frequently admitted to critical care units [63]. Our results reveal that hypernatremic patients receiving chemotherapy were not at risk of developing VTE, which may be attributed to this high-risk population receiving thromboprophylaxis treatment. The guidelines on VTE prevention in oncology from the United States National Comprehensive Cancer Network [64, 65] and the American Society of Clinical Oncology [66] suggest that thromboprophylaxis should be considered for high-risk ambulatory patients with cancer who receive chemotherapy.

The limitations of this work include a current shortage of biological data on the plausibility of the link between hypernatremia and postoperative thromboembolism. The association between hyponatremia and pulmonary embolism has been established. However, this association has not been established for deep vein thrombosis. Second, in the ACS NSQIP database, only one pre-operative serum sodium value is available and there was large variability in the collection and timing of preoperative blood work. However, sensitivity analyses suggested that risks were similar in patients with sodium measurements taken within 1 week of surgery. Third, patients are followed after surgery for 30 days thus omplications or death after that period are not included. Fourth, around 260,123 (19.0%) individuals out of 1,368,827 were excluded due to missing values on sodium levels. This selection bias has not been accounted for in this study.. Fifth, patients with severe cases of both hyponatremia and hypernatremia likely received some form of treatment. Thus, the incidence of hyponatremia and hypernatremia reported in our study may not be representative of the rates on the day of surgery. Finally, since this study aims at studying causality between sodium imbalances and VTE using a large retrospective dataset, much confounding remains unaddressed despite our careful adjustment for many clinically and statistically relevant factors. For instance, we cannot exclude the possibility of unmeasured confounding factors such as the use of diuretics and DVT prophylaxis, which is unavailable in the ACS NSQIP database analyzed. Thus, it is still not clear whether the associations between both hyponatremia and hypernatremia and increased risk of VTE, morbidity and mortality are due to the adverse effects of sodium imbalances or the underlying diseases. Future work needs to be performed to establish whether sodium imbalance is in fact a causal factor for post-operative VTE.

## Conclusion

Our results reveal an increased VTE risk, as well as increased mortality and morbidity, in both hyponatremic and hypernatremic patients. Because sodium levels are routinely measured in hospitalized patients, they could be easily utilized in identifying patients at risk of developing VTE. Recent evidence suggests that an improvement in serum sodium in hyponatremic patients is associated with a reduction of overall mortality [67]. Thus, hypernatremia and hyponatremia in surgical patients should not be ignored and patients with sodium imbalances ought to be more closely monitored for potential complications after surgery.

## Appendix

**Table 6:** Clinically relevant potential confounders

	VTE	Mortality	Wound	Cardiac	Respiratory	Urinary	CNS	Sepsis	Bleeding	Composite Morbidity	Return OR	Readmission
Age	X	X		X		X	X	X				X
Gender				X								
Race	X											
Type of Anesthesia	X	X		X								
ASA Class		X		X	X						X	X
Infected Surgical Wound Class		X	X								X	X
Mean Total Operation Time min	X											
Intraop Transfusions	X	X	X	X	X			X				
CHF w/in 30 days	X	X		X	X							X
MI w/in 6 months	X	X		X								
History of angina		X		X								
Tobacco use in past year	X	X		X	X		X			x		
History of severe COPD		X			X					x		X
Esophageal varices in previous 6 months		X							X	x		
Acute renal failure	X	X										
Currently on dialysis		X		X	X			X	X	X		X
Impaired sensorium in previous 48 h		X			X							
Hemiplegia	X	X										
History of transient ischemic attacks		X					X					
History of CVA with neurological deficit	X	X					X			X		
History of CVA without neurological deficit		X										
Tumor involving CNS	X	X					X					
Bleeding disorder		X							X		X	X
Weight loss > 10% in previous 6 months		X										
Disseminated cancer	X	X		X	X			X		X		X
Chemotherapy in previous 30 days	X											
BMI	X	X		X	X	X	X			X		X
Diabetic on oral drugs or insulin		X	X	X		X	X	X		X		
Hypertension requiring meds		X		X			X					
Peripheral vascular disease		X	X									
Rest pain or gangrene	X	X						X				
Open wound (with or without infection)			X					X			X	X
Steroid use for chronic condition	X							X				
Systemic sepsis in previous 48 h		X										
WBC count								X				
Platelet count	X	X					X					
INR									X			
Creatinine		X										
Serum albumin			X									
Emergency case		X	X	X								
Surgical Specialty	X	X										
Dyspnea		X			X							
Functional health status Prior to Surgery	X	X										
Ventilator dependent		X			X							
Current pneumonia		X			X			X				
Coma > 24 h	X	X										
Paraplegia	X											
Quadraplegia	X											
Bilirubin		X										
Hematocrit		X		X								
PTT									X			
Duration of Anesthesia		X		X	X							

## Abbreviations

ACS NSQIP: American College of Surgeons National Surgical Quality Improvement Program
ASA: American Society of Anesthesiologists
BMI: Body mass index
CNS: Central nervous system
ICD: International Classification of Diseases
IPCD: Intermittent pneumatic compression device
SCR: Surgical Clinical Reviewer
VTE: Venous thromboembolism
